# On the understanding and feasibility of “Breakthrough” Osmosis

**DOI:** 10.1038/s41598-019-53417-6

**Published:** 2019-11-11

**Authors:** Jun Jie Wu, Robert W. Field

**Affiliations:** 1 Department of Engineering, Durham University, Durham, DH1 3LE UK; 2 Department of Engineering Science, University of Oxford, Oxford, OX1 3PJ UK

**Keywords:** Materials for devices, Energy science and technology, Chemical engineering

## Abstract

Osmosis is the movement of solvent across a permselective membrane induced by a solute-concentration gradient. Now in ‘Forward Osmosis’ it is empirically observed that the diffusion of the solute is counter to that of the solvent i.e. there is so-called “reverse salt diffusion”. However it has been recently suggested, in a theoretical paper, that if allowance is made for minor deviations from ideal semi-permeability then operation in an overlooked mode of “breakthrough” osmosis would be possible and importantly it would yield relatively large rates of osmosis. A consequential prediction was that in “breakthrough mode”, Pressure-Retarded Osmosis (PRO) would generate very high power densities exceeding those in the conventional mode by one order of magnitude. The practicality of this suggestion was explored and necessarily questions were then raised regarding the foundation of the Spiegler-Kedem-Katchalsky model.

Arising from: Yaroshchuk, A., *Sci. Rep.* 7, 45168 (2017); 10.1038/srep45168

## Introduction

Great interest is being shown in processes with the potential to produce clean sustainable energy. Unlike solar and wind energy, which are both dependent on availability and the weather, the Blue Energy processes based upon the controlled mixing of seawater and fresh water have the advantage that they can be operated continuously. One process is Pressure-Retarded Osmosis (PRO), which has received renewed attention over the last two decades and was the subject of a recent thought-provoking theoretical study^1^. Following a contextual introduction on Blue Energy, Yaroshchuk’s recent model^1^, which is an application of the Spiegler-Kedem-Katchalsky (SKK) model to pressure retarded osmosis (PRO) is analysed with his boundary conditions. The mathematics of the derivation are sound but doubts are raised about the boundary conditions used and the validity of the underlying SKK model.

The SKK model is a well-established model for reverse osmosis. Now as Forward Osmosis (FO) is simply osmosis, it might be thought that the SKK model could be applied without reservation to FO and by extension to pressure retarded osmosis (PRO) as in^1^. However, in investigating the possibility and practicality of employing tailored PRO membranes to generate very high power densities, the foundations of the SKK model were found to be questionable. This was an unexpected finding. In parallel to this investigation, we chose to take the model in^1^ at face value and translated the analytical dimensionless analysis into an engineering science analysis, based on the use of practical values for the parameters. It was found that for practical salinities of the draw solution the outcomes are constrained such that the impact arising from the inclusion of a “leakiness” parameter is minor.

The second Blue energy process is Reverse Electrodialysis (RED) which is being pioneered in the Netherlands. With RED, the salinity gradient between seawater and river water is exploited via a stack of alternating cation and anion exchange membranes, and due to the chemical potential difference between salt and fresh water a voltage is generated across each membrane. A 50 kW pilot plant on the Afsluitdijk causeway in the Netherlands was opened in late 2014^2^. The aim of this pilot is to assess the technical feasibility of RED under real-life conditions using fresh Ijsselmeer water, which is on one side of the causeway and salt water from the Wadden Sea which is on the other side. Wetsus, who led the way on RED, remain research active in this area^3,4^.

With RED, electricity is generated directly from the membrane stack but PRO uses the osmotic pressure difference between a saline stream and fresh water to produce osmotic flow, which together with an appropriate back pressure, is used to drive a hydro turbine. The conceptual simplicity of PRO was recognised by the pioneers of reverse osmosis^5^ but concern about costs has always been a problem^6–8^. Nevertheless the desire to develop renewable energy options and the step changes in membrane development has led to PRO receiving significant attention in the past 15 years. The first pilot osmotic power plant was opened by the Norwegian energy company Statkraft in 2009 and it was reported the following year that the Statkraft prototype was designed to generate 10 kW of power and that the company planned to build a full-scale 25 MW osmotic power plant by 2015^9^. However the pilot plant closed in 2014 due to the low power density (power per unit of membrane area) of the prototypes and the questionable economic feasibility of the process. Indeed at its peak, the facility had produced power of just 2–4 kW before being closed. Despite the company’s efforts, they concluded that within the current market outlook the technology could not be sufficiently developed to become competitive “within the foreseeable future”^10^. Today it is generally accepted that if PRO is to be commercially viable then it will be necessary to use resources with a higher salinity than seawater, for example brine from a reverse osmosis desalination plant^11,12^. While this would tend to increase the osmosis and permit a higher backpressure, both of which lead to higher power densities, there is the inhibiting effect of a relatively high salt concentration within the support layer, due to the flux driven phenomenon known as internal concentration polarisation (ICP). This acts to reduce the difference in salinity across the active layer of the membrane and thereby dampen the positive effects of increased draw salinity^13^. Ideally a doubling of draw salinity would lead to a four-fold increase in power density but the mass transfer boundary layers, both external and ICP, have a negative impact. Thus it is generally accepted that increases in power density with increasing draw salinity will be more modest^13,14^.

A recent thought provoking theoretical paper applied the SKK model and deduced a contrary position^1^. It was contended that non-ideally semi-permeable supported membranes could be operated in an overlooked “breakthrough” mode in which ICP ceases to be important. It is claimed that one consequence is that non-ideally semi-permeable supported membranes could be operated, under certain conditions, with no reverse solute diffusion. A consequential outcome would be that ICP will be minimised and so it should be technically possible for certain PRO processes to achieve unusually high power densities; densities that would exceed by one order of magnitude those achieved previously. Secondly, it was suggested that much more robust support layers could be used without thicker supports having a detrimental effect upon flux.

The basis of the innovative theoretical analysis is reviewed and consideration given as to whether it can be extended beyond the purely analytical. Even though there was no experimental confirmation, it was clearly suggested^1^ that “breakthrough” could involve solute flux sign reversal (i.e. a transition from counter-current to co-current fluxes) and that the “dramatic change in the behaviour is ultimately caused by the change in the direction of solute flow through the membrane”. Whilst this specific possibility is rebutted, the prospect of the SKK model giving rise to an enhanced fluxes is evaluated.

Before moving to the Results section, it is noted that the SKK three-parameter model^15^ has previously been used to describe the mass transfer across the active layer of a FO/PRO membrane^16^. In the modelling of their experimental results, they found little difference between the standard solution-diffusion (S-D) model and the predictions of the SKK model for which a value of 0.92 was appropriate for the reflection coefficient (σ). Appropriately the modelling of the experimental work made due allowance for mass transfer on either side of the active layer. No evidence of any “breakthrough” was reported but the degree of ‘leakiness’ was greater than the desired value suggested in^1^. Secondly we note that whilst irreversible thermodynamic arguments were used to derive the solute and solvent transport equations of the SKK model the membrane itself is treated as a “black box”^17^. The values of the three parameter are empirically determined and, in all probability, depend upon the solute concentration in the barrier layer. Thus the values determined for RO will not apply for FO applications. Later in the paper, models in addition to the SKK model are briefly explored to check whether other models predict “breakthrough”.

## Results

**Figure 1:**
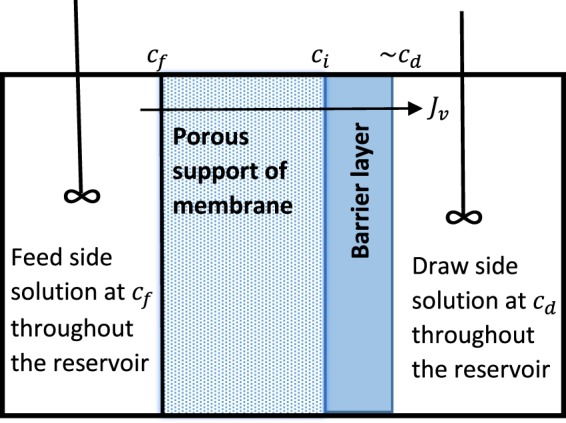
Supported membrane with idealised mass transfer conditions envisaged in the feed and draw reservoirs; both taken to be perfectly stirred. There is a constraint that at steady state any net component flux through the porous support layer will be identical to that through the barrier layer.

The  symbols are essentially the same as those used in^1^. No fault has been found with the mathematical derivations *per se* made in^1^, rather it is certain assumptions and the soundness of the underlying SKK model that is questioned, and these matters are addressed later. A schematic of the system being considered is given in Fig. 1.

The model in^1^, is now analysed as is, with the same boundary conditions which include *c*_*d*_ = *c*_*m*_. This is equivalent to stating that $P{e}_{bl}\to 0$. Hence

1 $$\frac{{c}_{i}\,\exp (-P{e}_{s}\,)-{c}_{f}}{1-\exp (-P{e}_{s})}=(1-\sigma )\cdot \frac{{c}_{d}\,\exp (-P{e}_{m}(1-\sigma ))-{c}_{i}}{1-\exp (-P{e}_{m}(1-\sigma ))}=(-\frac{{J}_{s}}{{J}_{v}})$$

where *Pe*_*m*_ is the Peclet number for the active layer of the membrane, *Pe*_*s*_ is the Peclet number for the support layer and other terms have been defined. Below components of Eq. 1 are referred to individually. Thus they are listed:

1a $$\frac{{c}_{i}\,\exp (-P{e}_{s})-{c}_{f}}{1-\exp (-P{e}_{s})}=(-\frac{{J}_{s}}{{J}_{v}})$$

1b $$(1-\sigma )\cdot \frac{{c}_{d}\,\exp (-P{e}_{m}(1-\sigma ))-{c}_{i}}{1-\exp (-P{e}_{m}(1-\sigma ))}=(-\frac{{J}_{s}}{{J}_{v}})$$

Now using Eq. (1a) to obtain an expression for ${c}_{i}$, the concentration difference across the active layer can be written as:

2 $${c}_{d}-{c}_{i}={c}_{d}-{c}_{f}\,\exp (P{e}_{s})-(-\frac{{J}_{s}}{{J}_{v}})[\exp (P{e}_{s})-1]$$

Having introduced Eq. (1a) it is appropriate to note that with a solute free feed (*c*_*f*_ = 0, the term (−*J*_*s*_/*J*_*v*_) is positive under all conditions *i*.*e*. there is counter-current flow of solute and solvent. Given that there is no solution giving co-current flow of solute and solvent for a salt-free feed, it is illogical to suppose that the addition of salt to the feed would, for any combination of physical properties and draw concentration, lead to a switch from counter-current flow to co-current flow.

Consequently it is necessary to part company from^1^ by not assuming that the SKK model is valid when it predicts co-current flow of solute and solvent. One recalls that whilst irreversible thermodynamic arguments were used to derive the solute and solvent transport equations of the SKK model, the membrane itself was treated as a “black box”^17^.

Our analysis of the potential influence of the inclusion of the third membrane parameter has been made using dimensional values of the membrane parameters. Thus the solvent fluxes are presented in units of µm/s and the draw solution in molarity with a range of concentrations that acknowledge the solubility limit of sodium chloride in water; at ambient conditions it is around 6.15 M. In this paper only CTA membranes are considered. The results labelled as SKK curves were obtained by the procedures outlined in Methods.

**Figure 2:**
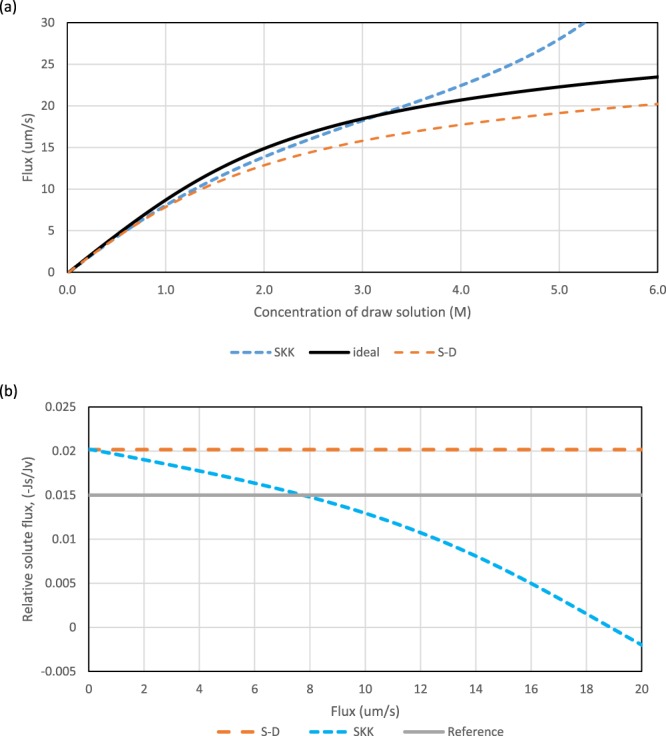
Predicted influence of draw concentration, *c*_*d*_, upon solvent flux, *J*_*v*_, and the consequential variation of relative solute flux $$(-\frac{{J}_{s}}{{J}_{v}})$$ with flux. Standard conditions except $${k}_{bl}\to \infty \,$$(i.e. $$P{e}_{bl}=0).$$ *S* = 350 µm.  See Table 1 for standard conditions. (**a**) predicted $${J}_{v}-\,{c}_{d}$$ curves for three models, S-D, SKK and ‘ideal’ (*B* = 0). (**b**) Variation of $$(-\frac{{J}_{s}}{{J}_{v}})$$ with *J*_*v*_ for S-D and SKK models. The ‘reference’ is: $$-{J}_{s}/{J}_{v}={c}_{f}$$.

Figure 2 includes a SKK curve that faithfully follows Yaroshchuk’s model^1^. The difference ${c}_{d}-{c}_{i}$ is eliminated by recognising (as in^1^) that the transmembrane solvent flux is (for the special case of ${c}_{d}={c}_{m}$) also given by:

3 $${J}_{v}=\sigma A\nu RT({c}_{d}-{c}_{i})$$

In summary it will be readily appreciated that the three Eqs 1a,1b and 3 contain three unknowns: $(\frac{-{J}_{s}}{{J}_{v}})$, *c*_*i*_, and either the flux *J*_*v*_ (if *c*_*d*_ is known) or *c*_*d*_ if flux is specified. Having established the set of concentrations, ${c}_{f},\,\,{c}_{i},\,{c}_{d}$, either of Eqs 1a to 1b can be used to find the predicted solute flux, *J*_*s*_. (As a consistency check, both should be calculated.) If the value of *J*_*s*_ is negative, it is considered to be a feasible value; otherwise the solution is considered physically invalid. This is where we part company with^1^ in terms of methodology.

For Fig. 2 the boundary conditions are those of^1^ and the draw side is taken to be an ideally stirred reservoir. The deviation of the SKK curve from the S-D curve becomes apparent only when the predicted relative solute flux is less than the reference value which corresponds to *c*_*f*_. When the SKK curve falls below this ‘reference’ the predicted reverse solute flux is less than the inlet convective influx of solute. Consequentially the predicted convective flux through the membrane is greater than the diffusive flux of solute from the draw towards the feed. The dramatic and alluring behaviour reported elsewhere^1^ only manifests itself when there is a prediction of co-current fluxes. This occurs around a flux of 18.5 µm/s but the physical possibility of such behaviour has been discount. In the prior region where the SKK model predicts counter-current fluxes, it and the S-D model give essentially the same values of *J*_*v*_ for the same value of draw concentration.

**Figure 3:**
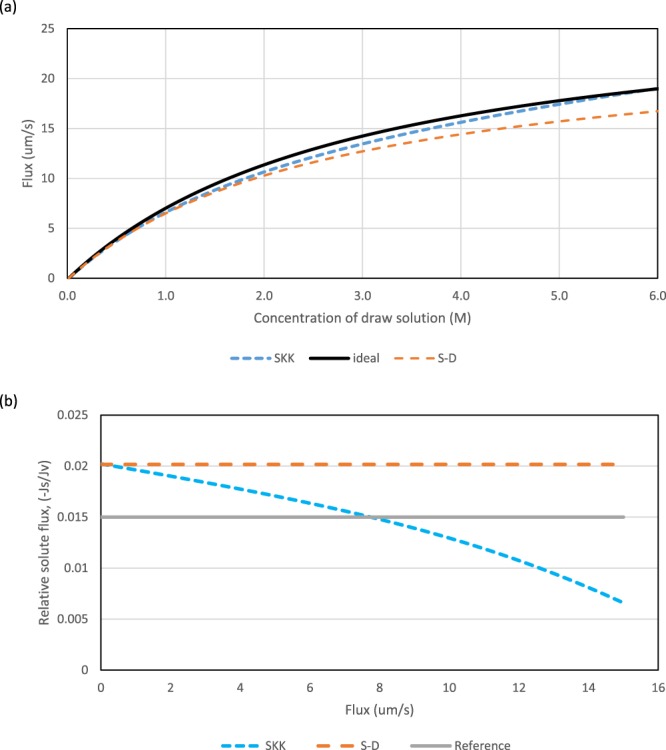
Predicted influence of draw concentration, *c*_*d*_, upon solvent flux, *J*_*v*_, and the consequential variation of relative solute flux $(-\frac{{J}_{s}}{{J}_{v}})$ with flux. *S*tandard conditions with *S* = 350 µm. See Table 1 for standard conditions. (**a**) predicted ${J}_{v}-\,{c}_{d}$ curves for three models, S-D, SKK and ‘ideal’ (*B* = 0). (**b**) Variation of $(-\frac{{J}_{s}}{{J}_{v}})$ with *J*_*v*_ for S-D and SKK models. The ‘reference’ is: $-{J}_{s}/{J}_{v}={c}_{f}$.

Claims of a major scientific advance should ideally be experimentally verified, or as a minimum, demonstrated through calculation using practical values. Thus we have extended the analysis in^1^ to include the draw side mass transfer boundary layer because as fluxes increase it is unreasonable to ignore it. The calculation procedure is outlined under Methods. For Fig. 3 the value of the draw side mass transfer coefficient is moderately large. Apart from this change, the basis of Fig. 3 is identical to that of Fig. 2. Figure 3 indicates that a degree of ‘leakiness’ (*i*.*e*.σ close to but not equal to unity) brings no discernible benefits.

**Figure 4:**
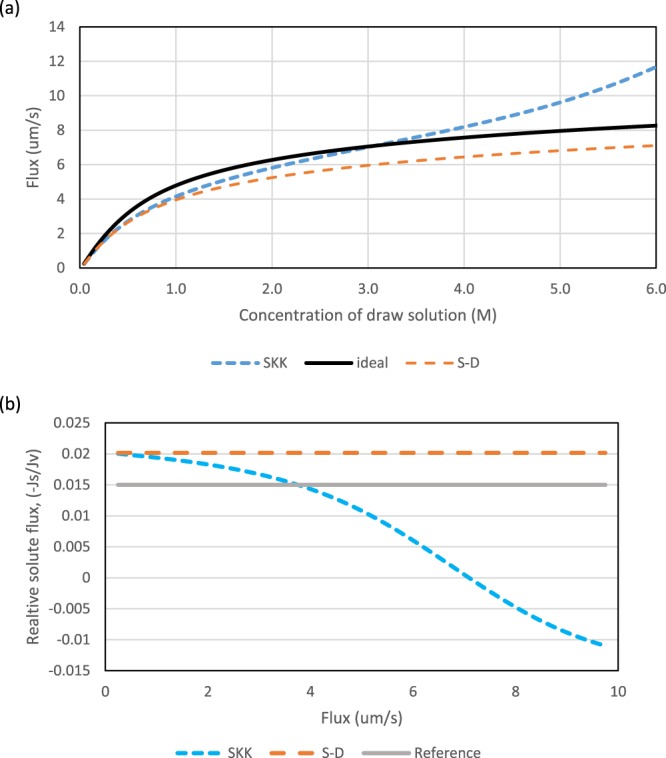
Predicted influence of draw concentration, *c*_*d*_, upon solvent flux, *J*_*v*_, and the consequential variation of relative solute flux $(-\frac{{J}_{s}}{{J}_{v}})$ with flux. *S*tandard conditions with *S* = 1000 µm. See Table 1 for standard conditions. (**a**) predicted ${J}_{v}-\,{c}_{d}$ curves for three models, S-D, SKK and ‘ideal’ (*B* = 0). (**b**) Variation of $(-\frac{{J}_{s}}{{J}_{v}})$ with *J*_*v*_ for S-D and SKK models. The ‘reference’ is: $-{J}_{s}/{J}_{v}={c}_{f}$.

A comparison of Figs 3a and 4a shows that the introduction of thicker support layers has a negative impact. The fluxes with the SKK model are relatively higher than those with the S-D model for a given value of the structural parameter, *S*, but membranes with thicker support layers are predicted to have lower absolute fluxes than those with a typical value of *S* and reflection coefficients of unity (i.e. S-D model with *S* = 350 µm). Thus there is no gain in terms of flux in switching to thicker support layers, even if a certain degree of ‘leakiness’ can be introduced. Our modelling suggests that membrane manufacturers should aim for low *B/A* ratios in order to be close to the ‘ideal’ curves shown in Figs 3a and 4a.

Figures 2–4 include the relative solute flux (−*J*_*s*_/*J*_*v*_). They show that the envisaged “breakthrough” mode only occurs when this ratio becomes negative. Thus there is no “breakthrough” before the supposed solute flux reversal. As co-current flows are physically unrealistic, it can be concluded that there is no mode that will entail unexpectedly high fluxes and, if operated with back-pressure in the PRO mode, unexpectedly high power densities. In Figs 2a and 4a the continuation of the SKK curves above the ‘ideal’ curve have been included as a mere hypothetical prediction so as to make the link with^1^. Even if such solutions had been possible, the corresponding fluxes are not high when draw side mass transfer is included even for a feed salinity of 0.015 M, which would be a very low value of salinity in any practical application.

With regard to the feedside boundary condition, it is noted that in^1^, a claim is made concerning very robust support layers. Namely “that in this [breakthrough] mode the osmotic flow becomes insensitive to the properties of membrane support (the curves calculated for different support properties converge). Therefore, the limiting case of infinitely thick supports …. is applicable also for finite (and even rather small) values of [thickness] *once the breakthrough mode is well established* [our emphasis].” Most importantly, the caveat “once the breakthrough mode is well established” is emphasised because if breakthrough is impossible then the imagined benefits of thick supports will not be realisable.

## Concluding Remarks

The supposed “breakthrough” mode would only occur if it were possible to engineer a reversal in the direction of the solute flux but theoretically, as shown herein, it is impossible. Notwithstanding this finding and our consequent concerns regarding the application of the SKK model to FO, the relevant equations of the SKK model (based on^1^) were solved to evaluate whether the impact of imperfections could be significant prior to the predicted reversal in the direction of the solute flux. For typical membrane parameters the impact is insignificant.

Owing to the fact that the point of zero net solute flux is a thermodynamic impossibility, the prediction of a region of positive solute flux must be viewed as an artefact of the SKK model when it is implemented with concentration and pressure invariant parameters. Use of other models of membrane transport, as discussed in Methods, indicate that FO and PRO cannot involve co-current flow of solute and solvent. If a new region had been discovered it would have provided a fresh impetuous to PRO research. More particularly, it would have stimulated experimental research to determine whether the claims could be verified experimentally. Alas this seems not to be worthwhile. Overall the situation regarding the potential of FO and PRO remains unchanged with the conclusions in^14^ remaining essentially as valid today as in 2013.

FO and PRO are the simplest of settings for the application of the Spiegler-Kedem-Katchalsky model. That this model has been shown to yield irrational results indicates that some detail in the SKK model is wrong. Now the equation for the salt flux includes the term $(1-\sigma )c{J}_{v}$ where c is the reference (virtual) solute concentration (as given in^18^ and used in^1^), σ is the solute reflection coefficient, and *J*_*v*_ is the solvent flux. In reverse osmosis (RO), this term can be linked to a *pressure* contribution because the solvent flux *J*_*v*_ is strongly influenced by feedside pressure in RO. Indeed in an alternative model, the solution-diffusion-imperfection model^18^, the term $(1-\sigma )c{J}_{v}$ is explicitly replaced by the term *L*_*i*_*c*_*d*_∆*P* (see Methods section) and there are no absurdities when this model is applied to FO. This suggests that the fundamental fault with the SKK model, when applied to FO, is linked to the use of the term $(1-\sigma )c{J}_{v}$ where $\sigma$ is assumed to be independent of both the solute concentration in the barrier layer and the hydraulic pressure difference across it. Notwithstanding the utility that the SKK model has demonstrated over a number of decades, it seems to have a fundamental shortcoming.

## Methods

Firstly we record the procedures adopted in making the calculations. The equations below do not assume that ${k}_{bl}\to \infty \,$(i.e. $P{e}_{bl}=0$) because in general one needs to consider a finite mass transfer coefficient and ${c}_{m} < {c}_{d}$, but they can be readily adapted for the special theoretical case of ${c}_{m}={c}_{d}$. Also the discussion of the boundary conditions has established a limitation, namely that co-current fluxes of solvent and solute are impossible. In^1^, equations (10) and (13) therein were combined to give equation (15). However a more appropriate form of the comprehensive equation needs to explicitly retain the ratio $-\frac{{J}_{s}}{{J}_{v}}$ because the value of this term needs to be checked, given our observation that co-current fluxes of solvent and solute are impossible i.e. the value of $-\frac{{J}_{s}}{{J}_{v}}$ cannot be negative.

4 $$\frac{{c}_{i}\,\exp (-P{e}_{s})-{c}_{f}}{1-\exp (-P{e}_{s})}=(1-\sigma )\cdot \frac{{c}_{m}\exp (-P{e}_{m}(1-\sigma ))-{c}_{i}}{1-\exp (-P{e}_{m}(1-\sigma ))}=\frac{{c}_{d}-{c}_{m}\exp (P{e}_{bl})}{\exp (P{e}_{bl})-1}=(-\frac{{J}_{s}}{{J}_{v}})$$

where *Pe*_*m*_ is the Peclet number for the active layer of the membrane, *Pe*_*s*_ is the Peclet number for the support layer and other terms have been defined. For ease of reference the individual components are:

4a $$\frac{{c}_{i}\exp (-P{e}_{s})-{c}_{f}}{1-\exp (-P{e}_{s})}=(-\frac{{J}_{s}}{{J}_{v}})$$

4b $$(1-\sigma )\cdot \frac{{c}_{m}\exp (-P{e}_{m}(1-\sigma ))-{c}_{i}}{1-\exp (-P{e}_{m}(1-\sigma ))}=(-\frac{{J}_{s}}{{J}_{v}})$$

4c $$\frac{{c}_{d}-{c}_{m}\exp (P{e}_{bl})}{\exp (P{e}_{bl})-1}=(-\frac{{J}_{s}}{{J}_{v}})$$

Now from Eq. (4a and 4c), the concentration difference across the active layer can be written as:

5 $${c}_{m}-{c}_{i}={c}_{d}\exp (-P{e}_{bl})-{c}_{f}\,\exp (P{e}_{s})-(-\frac{{J}_{s}}{{J}_{v}})[\exp (P{e}_{s})-\exp (-P{e}_{bl})]$$

The second equation involving this difference is:

6 $${J}_{v}=\sigma A\nu RT({c}_{m}-{c}_{i})$$

An explicit equation linking the solvent flux and the draw concentration was derived from Eqs 4–6 to generate the SKK curves in Figs 2–4. The outline follows. The following terms are defined:

7 $$G=\frac{1-\sigma }{1-\exp (-F)}\,{\rm{where}}\,F=P{e}_{m}(1-\sigma )$$

One can rewrite and expand Eq. 4b as follows:

8 $$(\,-\,\frac{{J}_{s}}{{J}_{v}})=G{c}_{m}\exp (\,-\,F)-G{c}_{i}=G({c}_{m}-{c}_{i})+G{c}_{m}(\exp (\,-\,F)-1)$$

Combining Eq. (4c) with Eq. (8) to eliminate *c*_*m*_ yields:

9 $$G({c}_{m}-{c}_{i})+G(\exp (\,-\,F)-1){c}_{d}\,\exp (\,-\,P{e}_{bl})=(\frac{-{J}_{s}}{{J}_{v}})[1\,-G(\exp (\,-\,F)-1)(\exp (\,-\,P{e}_{bl})-1)]$$

Equations 5 and 9 can be combined to eliminate the term $(\frac{-{J}_{s}}{{J}_{v}})$. The term (*c*_*m*_ − *c*_*i*_) is then eliminated from the combined equation through use of Eq. (6). The resultant equation enables one to find *c*_*d*_ explicitly for given values of *J*_*v*_ (whose value also specified all of the Peclet numbers), *c*_*f*_, and fixed system and membrane parameters including *k*_*bl*_, *c*_*f*_, S/D etc. Solutions were obtained using Microsoft Excel. The values used for standard conditions are given in Table 1. (In our analysis there was a perfectly stirred reservoir supplying the support layer).

**Table 1:** Standard conditions for generation of flux-draw concentration curves.

Parameter	Hydraulic permeability	Solute permeability	Reflection coefficient	Structural parameter	Draw side mass transfer coefficient	Feed side
Symbol (units)	A (µm s^−1^ MPa^−1^)	B (µm s^−1^)	σ	S	${k}_{bl}$ (µm s^−1^)	${c}_{f}$ (M)
Value	2	0.2	0.99	varied	30	0.015
Source/comment	^13^	^13^	^1^	Minimum as^13^	About 4x greater than value found for current modules^22^	Very modest salinity

Secondly, three other models are now briefly examined to see whether any would entail a “breakthrough” mode. The solution-diffusion model has been widely applied^17^ but it would not lead to the prediction of any “breakthrough”. Indeed if one assumes that the osmotic pressure follows the van’t Hoff equation, this model predicts a fixed ratio between the solute and solvent fluxes independent of feed and draw concentrations^19^:

10 $$(\,-\,{J}_{s}/{J}_{v})=\frac{B}{A\beta {R}_{g}T}$$

where β is the van’t Hoff coefficient, ${R}_{g}$ is the universal gas constant, and *T* is the absolute temperature. Clearly the fluxes remain counter-current.

The solution-diffusion-imperfection model (SDI)^20^ was formulated to address some of the shortfalls in the basic solution-diffusion model^17^. The key equations for the barrier layer, when applying it to PRO, would be:

11 $${J}_{v}=A(\Delta P-\Delta \pi )+{L}_{i}\Delta P$$

12 $${J}_{s}=B({c}_{d}-{c}_{i})+{L}_{i}{c}_{d}\Delta P$$

where ${L}_{i}$ is the permeability of imperfections, ∆*P* is the transmembrane pressure difference, $\Delta \pi$ is the osmotic pressure difference across the membrane and other symbols have their normal meaning with *A* being the water permeance and B the solute permeance through the barrier layer. Clearly for PRO this model suggests an augmentation of the solute flux (compared with the S-D model) in the direction *away* from the pressurised draw solution; see Eq. (12). For FO operation the pressure difference ($\Delta P$) would be zero and the SDI model reverts to the basic solution-diffusion model. So for osmotically driven processes, the SDI model would never entail a decrease in the ratio $(\,-\,{J}_{s}/{J}_{v})$ and the fluxes always remain counter-current for FO and PRO.

Additionally consideration was given to the applicability of a pore flow model^21^. However one needs perm-selectivity to maintain a osmotic flow from feed to draw and a channel has no interfaces between the bulk solutions and the pore fluid. A channel with pore flow would have a convective term and for PRO this would lead to an augmentation of the solute and solvent flux in the direction away from the pressurised draw solution. For FO operation, there would essentially be no convective component as the pressures would be the same on both sides. The diffusive components would be counter-current with water diffusing towards the draw and salt diffusing towards the opposite side, which is of lower salinity i.e. counter-current fluxes.

A summary of osmotically driven flow models is given in Table 2.

**Table 2:** Summary of membrane models with osmotically driven solvent flux.

Model type	Variation of $(\frac{-{{\boldsymbol{J}}}_{{\boldsymbol{s}}}}{{{\boldsymbol{J}}}_{{\boldsymbol{v}}}})$ with flux	Direction of solute flux	Predicted variation of solvent flux with *c*_*d*_
Spiegler-Kedem-Katchalsky as implemented in^1^	Declines	Possibility of co-current flow is predicted	Before point at which $(\frac{-{{\boldsymbol{J}}}_{{\boldsymbol{s}}}}{{{\boldsymbol{J}}}_{{\boldsymbol{v}}}})$ is predicted to reach zero, augmentation modest compared with S-D
solution-diffusion	Remains constant	Always counter to solvent flux	Logarithmic
solution-diffusion-imperfection	Remains constant for FO.	Always counter to solvent flux	Logarithmic

## References

[1] Yaroshchuk A (2017). “Breakthrough” osmosis and unusually high power densities in Pressure-Retarded Osmosis in non-ideally semi-permeable supported membranes. Sci. Rep..

[2] https://www.dutchwatersector.com/news-events/news/12388-dutch-king-opens-world-s-first-red-power-plant-driven-on-fresh-salt-water-mixing.html Accessed September 6th 2018.

[3] Moreno J, Slouwerhof E, Vermaas DA, Saakes M, Nijmeijer K (2016). The Breathing Cell: Cyclic Intermembrane Distance Variation in Reverse Electrodialysis. Environ. Sci. Technol..

[4] Wetsus Annual Report, 2016, at (2016).

[5] Loeb S (1976). Production of energy from concentrated brines by pressure retarded osmosis. I. Preliminary technical and economic correlations. J. Membr. Sci..

[6] Loeb S, Norman RS (1975). Osmotic Power Plants. Science.

[7] Lee KL, Baker R, Lonsdale H (1981). Membranes for power generation by pressure-retarded osmosis. J. Membr. Sci.

[8] Loeb S (2002). Large-scale power production by pressure-retarded osmosis using river water and sea water passing through spiral wound modules. Desalination.

[9] Achilli A, Childress AE (2010). Pressure retarded osmosis: From the vision of Sidney Loeb to the first prototype installation — Review. Desalination.

[10] http://www.powermag.com/statkraft-shelves-osmotic-power-project/ Accessed 27th October 2017.

[11] Straub AP, Deshmukh A, Elimelech M (2016). Pressure-retarded osmosis for power generation from salinity gradients: is it viable?. Energy Environ. Sci..

[12] Lin S, Straub AP, Elimelech M (2014). Thermodynamic limits of extractable energy by pressure retarded osmosis. Energy Environ. Sci..

[13] Field RW, Wu JJ (2018). On boundary layers and the attenuation of driving forces in forward osmosis and other membrane processes. Desalination.

[14] Field RW, Wu JJ (2013). Mass transfer limitations in forward osmosis: Are some potential applications overhyped?. Desalination.

[15] Spiegler KS, Kedem O (1966). Thermodynamics of hyperfiltration (reverse osmosis): criteria for efficient membranes. Desalination.

[16] Attarde D, Jain M, Gupta SK (2016). Modeling of a forward osmosis and a pressure-retarded osmosis spiral wound module using the Spiegler-Kedem model and experimental validation. Separation and Purification Technology.

[17] Wang J (2014). A critical review of transport through osmotic membranes. J. Membr. Sci..

[18] Yaroshchuk AE (1995). Osmosis and reverse osmosis in fine-porous charged diaphragms and membranes. Adv. Colloid Interface Sci..

[19] Tang CY (2010). Coupled effects of internal concentration polarization and fouling on flux behavior of forward osmosis membranes during humic acid filtration. J. Membr. Sci.

[20] Yaroshchuk AE (1995). Solution-Diffusion-Imperfection model revised. J. Membr. Sci..

[21] Opong WS, Zydney AL (1991). Diffusive and through Convective Protein Transport Asymmetric Membranes. AIChE Journal.

[22] Field RW, Siddiqui FA, Ang P, Wu JJ (2018). Analysis of the influence of module construction upon forward osmosis performance. Desalination.

